# Interaction Between the Cannabinoid and Vanilloid Systems on Anxiety in Male Rats

**DOI:** 10.18869/nirp.bcn.8.2.129

**Published:** 2017

**Authors:** Nafiseh Faraji, Alireza Komaki, Iraj Salehi

**Affiliations:** 1. Neurophysiology Research Center, Hamadan University of Medical Sciences, Hamadan, Iran.; 2. Department of Biology, Hamadan Branch, Islamic Azad University, Hamadan, Iran.

**Keywords:** Anxiety, Elevated plus maze, CB1 receptors, TRPV1 receptors, Rat

## Abstract

**Introduction:**

Previous studies have shown that the cannabinoid system is involved in anxiety. In addition, transient receptor potential vanilloid type-1 (TRPV1) channels are new targets for the development of anxiolytics. The present study investigated the possible interaction between the cannabinoid and vanilloid systems on anxiety-like behavior in rats.

**Methods:**

Four different groups of male Wistar rats received intraperitoneal (IP) injections of (1) vehicle (DMSO+saline), (2) cannabinoid receptor agonist WIN55212-2 (WIN) (1 mg/kg), (3) TRPV1 receptor antagonist capsazepine (CPZ) (5 mg/kg), or (4) combined WIN (1 mg/kg) and CPZ (5 mg/kg) treatment 30 minutes before testing in the elevated plus maze.

**Results:**

The results showed that compared to the control (vehicle), both WIN and CPZ increased the time spent and number of entries on the open arms. Co-administration of WIN and CPZ had a synergistic effect, i.e., the number of entries and time spent on the open arms was greater than that in the groups administered the two compounds alone. The total distance travelled by rats and total number of entries on to the arms did not significantly differ between groups.

**Conclusion:**

Acute neuropharmacological blockade of the TRPV1 receptor or stimulation of the CB1 receptor produced an anxiolytic effect. It seems that antagonism of the vanilloid system modulates cannabinoid gain that rises the anxiolytic effect. TRPV1 antagonism may amend generation of endocannabinoids, which in turn increases anxiolytic impact. These results suggest that two systems could act on or share a common signaling pathway affecting the expression of anxiety.

## 1. Introduction

Anxiety disorders are the most common mental disorders in modern society (Di Marzo et al., 1998; Schneier, 2011). Many neurotransmitters such as the GABAergic, noradrenergic, and endocannabinoid systems modulate anxiety (Viveros, Marco, & File, 2005) through their actions in various brain regions (Herkenham et al., 1990). There is accumulating evidence that Cannabis sativa, acting via the cannabinoid system, modulates the expression of anxiety (Bergamaschi, Queiroz, Zuardi, & Crippa, 2011; Crippa et al., 2011; Hill and Gorzalka, 2004; Schneier, 2011). There have been numerous studies on the effects of the cannabinoid system in modulating anxiety but with conflicting results (Haller, Bakos, Szirmay, Ledent, & Freund, 2002; Marco and Viveros, 2008; Navarro et al., 1997; Patel and Hillard, 2006; Viveros, Marco, & File, 2005; Komaki, Abdollahzadeh, Sarihi, Shahidi, & Salehi, 2014a; Komaki et al. 2015a).

The endogenous cannabinoid system, also known as the endocannabinoid system, regulates the expression of anxiety (Ruehle, Rey, Remmers, & Lutz, 2012). Cannabinoid receptor agents have been shown to exert anxiolytic (Patel and Hillard, 2006; Saito, Wotjak, & Moreira, 2010) and anxiogenic effects (Carvalho and Van Bockstaele, 2012; Degroot, 2008, Haller et al., 2004a; Haller et al., 2004b; Moreira, Aguiar, & Guimarães, 2007; Moreira, Kaiser, Monory, & Lutz, 2008). Cannabinoid agents act through two receptors: CB1 and CB2 (Howlett et al., 2002). CB1 is the most abundant G-protein coupled receptor found in the mammalian brain (Matsuda, Lolait, Brownstein, Young, & Bonner, 1990) and are particularly abundant in the cortex, hippocampus, nucleus accumbens, and amygdala (Millan, 2003). The blockade and genetic deletion of the CB1 cannabinoid receptor modulate anxiety in mice (Haller, et al., 2002; Thiemann, Watt, Ledent, Molleman, & Hasenöhrl, 2009).

Transient receptor potential vanilloid-1 (TRPV1) receptor is a subfamily of vanilloid family (Morales-Lazaro Simon, & Rosenbaum, 2013). TRPV1 is expressed widely throughout the central nervous system and peripheral afferent fibers (Caterina, Schumacher, Tominaga, Rosen, & Levine, 1997; Chung, Lee, Yuichi, & Willis, 1985; Roberts, Davis, & Benham, 2004; Tóth, Blumberg, & Boczán, 2005). TRPV1 promotes neuronal depolarization, increasing their firing rate and synaptic activity (Xing and Li, 2007). It has been shown that TRPV1 also modulates anxiety behavior (Kasckow, Mulchahey, & Thomas, 2004; Marsch et al., 2007). Similar to the reports of cannabinoid modulation of anxiety, the effects of the vanilloid system on the expression of anxiety are controversial (Aguiar, Terzian, Guimarães, & Moreira, 2009; Mascarenhas, Gomes, & Nunes-de-Souza, 2013).

Previous studies reported that the co-expression of CB1 and TRPV1 receptors was observed in several brain regions (Cristino et al., 2006; Mezey, 2000; Sagar et al., 2004; Tóth, Blumberg, & Boczán, 2005). There are functional relationships between CB1 and TRPV1 receptors in the nervous system (Hermann et al., 2003; Kim, Bok, Chung, Chung, & Jin, 2009; Sagar et al., 2004). Further, the cannabinoid and vanilloid systems interact in the brain to modulate anxiogenic behavior (Aguiar et al., 2009; Fogaça, Aguiar, Moreira, & Guimarães, 2012; Moreira, Aguiar, & Guimarães, 2007; Rubino et al., 2007).

While data is available on the individual effects of the cannabinoid and vanilloid systems in the expression of anxiety, the simultaneous stimulation or inactivation of these two systems and their interactive mechanisms have not been studied for their effects on the expression of anxiety. Thus in this experiment, we intend to evaluate the hypothesis that the effects of the cannabinoid system on anxiety are mediated by the vanilloid system results, specifically through the activity of TRPV1 receptors. The interaction of these two neuromodulators in the modification of anxiety could have remedial significance in clinical situation. In the present experiment, we examined the consequences of co-administration of a cannabinoid agonist and a vanilloid antagonist on the expression of anxiety-like behaviors in rats.

## 2. Methods

### 2.1. Animals

Male Wistar rats (obtained from the Razi institute, Iran) weighing 200–250 g were used throughout these experiments. Animals were housed in groups of four per cage under a 12 h light/dark cycle (lights on at 07:00 h) at a constant temperature of 23±2°C and given ad libitum food and water. All experiments were carried out in a quiet room under controlled light conditions between 11:00 and 15:00. Animals were allowed to acclimate to vivarium conditions for 1 week prior to behavioral testing. The experimental protocols were approved by the Hamadan University of Medical Sciences and were conducted in accordance with the Guide for Care and Use of Laboratory Animals published by the United States National Institutes of Health (NIH Publication No. 85-23, revised 1985).

### 2.2. Drugs and administration

WIN 212-2 [(R)-(+)-[2,3-Dihydro-5-methyl-3-(4-morpholinylmethyl) pyrrolo [1,2,3-de]-1, 4-benzoxazin-6-yl]-1-napthalenylmethanone] (WIN) (Sigma-Aldrich,Germany) and capsazepine [N-[2-(4-Chlorophenyl)ethyl]-1,3,4,5-tetrahydro-7,8-dihydroxy-2H-2-benzazepine-2-carbothioamide] (CPZ) (Tocris, UK) were dissolved in dimethyl sulfoxide (DMSO; Sigma-Aldrich, Germany) + saline. The solutions were prepared freshly. The animals were randomly divided into the control group and 3 experimental groups. The control group (n=10) received vehicle (saline+DMSO 8%). All rats received intraperitoneal (IP) injections in a volume of 1 mL/kg 30 minutes prior to behavioral testing. One week after acclimatization, CPZ (n=10), WIN (n=10), or a combination of these drugs (WIN+CPZ) (n=10) were administered 30 minutes prior to behavioral testing. Drug doses were calculated based on preceding experiments: WIN: 1 mg/kg; IP (Richter and Löscher, 1994; Baek, et al., 2009; Burgos, et al., 2010; Fernández-López, et al., 2010; Shabani, et al., 2012; Komaki, et al., 2015a) and CPZ: 5 mg/kg; IP (Cabranes, et al., 2005; Nguyen, et al., 2010; Almeida, et al., 2014; Kaczocha, et al., 2015; Magierowski, et al., 2015).

### 2.3. Elevated Plus-Maze (EPM)

The apparatus consisted of two opposing open arms (50×10 cm each) without side walls, adjacent to two enclosed arms (50×10×50 cm each), with a central platform common to all arms (10×10 cm). The apparatus was elevated 50 cm above the ground (Pellow et al., 1985; Pellow and File, 1986). Thirty minutes after drug administration, each animal was placed in the center of the maze, facing one of the open arms. The rats were allowed to explore the maze, and their behavior was recorded for 10 minutes by using a digital camera mounted above the maze. The time spent and percentage of entries on to the open and enclosed arms were recorded. Distance travelled was calculated and used as a measure of locomotion during the 10 minutes test period (Lister, 1987; Ganji et al., 2016). After each test, the apparatus was cleaned with 10% ethanol to eliminate the remaining odors. Each animal was used only once.

### 2.4. Statistical analysis

The statistical analysis of data was performed by one-way analysis of variance (ANOVA) followed by Tukey post-hoc analysis. All the results are presented in terms of mean±SEM. A p value less than 0.05 was considered to be significant.

## 3. Results

### 3.1. Effects on the total distance covered by rats

**Figure 1.**
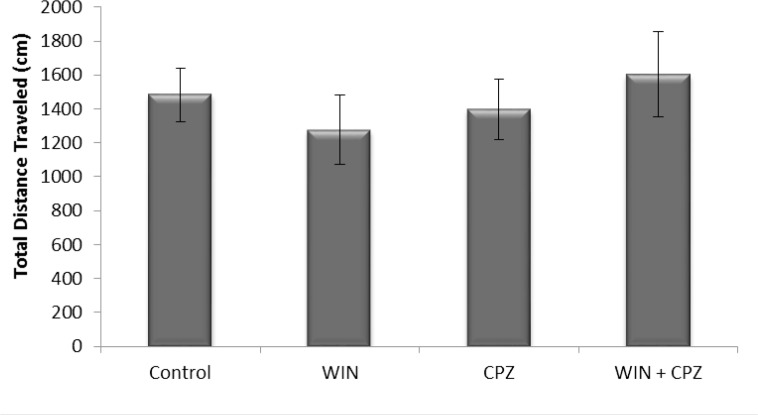
The effect of WIN212-2 (WIN), capsazepine (CPZ) and co-administration WIN+CPZ on the total distance covered by the rats during the 10 min test session (n=10 in each group). Data are presented as mean±SEM.

The total distance covered by the WIN, CPZ, and WIN+CPZ treated rats during the 10 minutes test period was not significantly different from controls (Figure 1).

### 3.2. Effects on the percentage of entries on to the open arms

**Figure 2.**
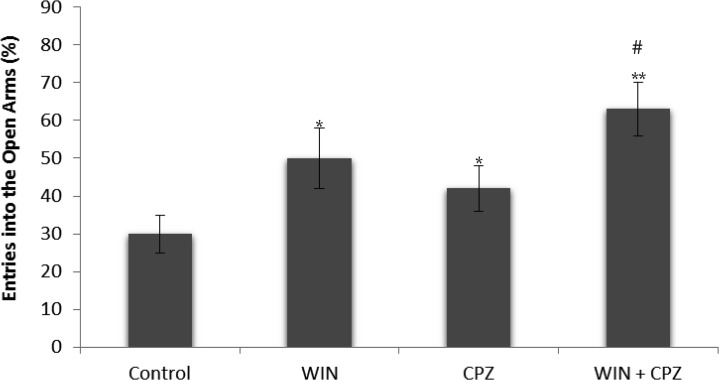
The effect of WIN212-2 (WIN), capsazepine (CPZ) and co-administration WIN+CPZ on the percentage of entries on to the open arms during the 10-min test session (n=10 in each group). Data are presented as mean±SEM. *: P<0.05, **: P<0.01 in comparison with control group. #: P<0.05 compared to the CPZ group.

The effects of WIN, CPZ, and WIN+CPZ on the percentage of entries on to the open arms are shown in Figure 2. One-way ANOVA revealed a significant difference between experimental groups in percentage of entries on to the open arms. Tukey’s post-hoc tests revealed significant increases in percentage of entries on to the open arms after administration of WIN, CPZ (P<0.05), and after the co-administration of WIN and CPZ (P<0.01) in comparison with the control group. Percentage of entries on to the open arms after co-administration of WIN and CPZ was significantly higher than the WIN and CPZ treated groups (P<0.05).

### 3.3. Effects on the time spent in open arms

**Figure 3.**
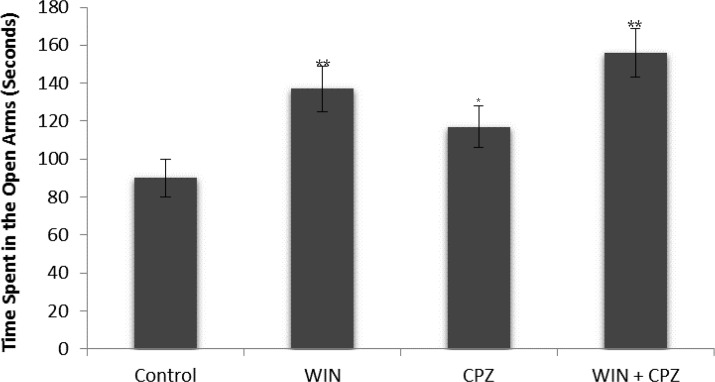
The effect of WIN212-2 (WIN), capsazepine (CPZ) and co-administration WIN+CPZ on the time spent in open arms during the 10 min test session (n=10 in each group). Data are presented as mean±SEM. *: P<0.05, **: P<0.01 in comparison with control group. #: P<0.05 compared to the CPZ group.

Compared to the control group, rats in the WIN group showed a significant decrease in the time spent on the open arms (P<0.01) whereas those in the CPZ (P<0.05) and WIN+CPZ (P<0.01) groups showed a significant increase. Further, compared to the rats in the CPZ group, those in the WIN+CPZ group showed a significant increase in the time spent on the open arms (P<0.05) (Figure 3).

### 3.4. Effects on the closed arms entry

**Figure 4.**
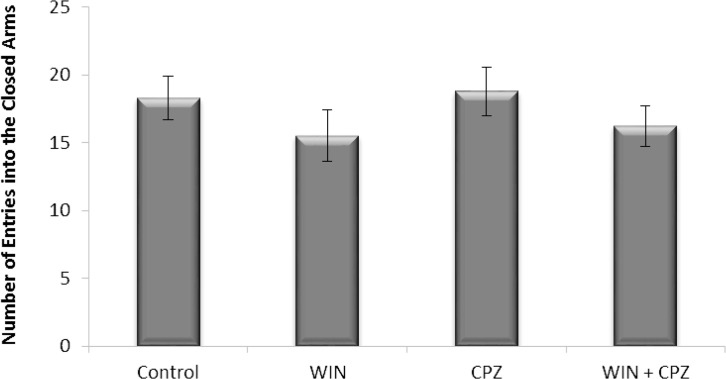
The effect of WIN212-2 (WIN) and capsazepine (CPZ) and co-administration WIN+CPZ on the number of closed arm entries during the 10 min test session (n=10) in each group). Data are presented as mean±SEM.

The number of entries on to the closed arms was not significantly different between WIN, CPZ, and WIN+CPZ groups compared to the control group (Figure 4).

## 4. Discussion

The results of the present study indicate that the CB1 agonist (WIN) and the TRPV1 antagonist (CPZ) show an anxiolytic profile in rats. Our findings demonstrated that WIN and CPZ increased both assigned values of the time spent and number of passage onto the open arena of the maze. The consequences of WIN and CPZ administrations were not associated with the changes in natural motor activity of the animals since the total distance traveled by the rats and the number of passages on to the enclosed arena did not change. Increase in the time and ratio of the passage on to the open arena in the absence of any change in locomotor action can be regarded as a capable index for anxiolytic activity of the substances (Pellow, Chopin, File, & Briley, 1985; Komaki et al., 2014b). In addition, co-administration of CPZ and WIN potentially increases the anxiolytic activity of these compounds in animals. Changes within the percentage of passage on to the open arena and time spent on these arena are regarded as reliable correlates of changes in the amount of anxiety in experimental models. Therefore, the EPM is advised as a validated and reliable device to reflect both anxiolyticand anxiogenic-like effects of various drugs (Pellow, et al., 1985; Pellow and File, 1986; Komaki, et al., 2014a; Komaki, et al., 2015b; Nemati, et al., 2015).

CB1 is a G-protein coupled receptor (Matsuda et al., 1990). Activation of CB1 receptors inhibits adenylate cyclase, resulting in decreases in intracellular calcium, and activates inwardly K^+^ channels (Howlett et al., 2002), reduces neurotransmitter release (Vaughan, Connor, Bagley, & Christie, 2000), and finally mediates anxiolytic-like effect (Rubino et al., 2007). Therefore, pharmacological substances that enhance endogenous cannabinoid signaling demonstrate anxiolytic-like actions (Bortolato et al., 2006; Kathuria et al., 2003).

Recent studies suggest the involvement of vanilloid receptors in the expression of anxiety-like behavior and the anxiolytic properties of cannabinoid agonists in mice and rats (Kasckow, Mulchahey, & Thomas, 2004; Patel and Hillard, 2006; Roohbakhsh, Moghaddam, Massoudi, & Zarrindast, 2007; Rubino et al., 2007). Blockade of TRPV1 resulted in anxiolytic-like behavior in TRPV1 receptor-deficient mice, (Marsch et al., 2007) and rats treated with a TRPV1 antagonist into the medial prefrontal cortex showed a similar effect (Aguiar et al., 2009). Moreover, blockade of TRPV1 channels induces anxiolytic-like effects (Aguiar et al., 2009; Fogaça et al., 2012). TRPV1 promotes cellular depolarization and increases neuronal firing rate (Xing and Li, 2007). TRPV1 is a calcium permeable ligand-gated cation channel that is expressed in various peripheral non-neuronal tissues and throughout the central nervous system (Montell et al., 2002). Activation of TRPV1 channels can stimulate calcium influx, facilitate the release of neurotransmitters (Musella et al., 2009, Starowicz et al., 2007) and may facilitate anxiogenic behavior (Marsch et al., 2007).

Different mechanisms are proposed to describe the interaction between the cannabinoid and vanilloid systems within the CNS. The attainable communication between TRPV1 and CB1 receptors on anxious status has been previously reported in hippocampus (Hakimizadeh and Oryan, 2012) or PAG (Campos and Guimaraes, 2009) regions. It has been suggested that lipid-based molecules such as anandamide-related compound may act on TRPV1 (di Marzo, et al., 1998; Beltramo and Piomelli, 1999), suggesting a possible interaction between the cannabinoid and vanilloid systems. Many studies have demonstrated that stimulation of CB1 and TRPV1 produces contrasting results in various research settings, including alteration in intracellular Ca^2+^ levels (Szallasi and Di Marzo, 2000) and glutamate liberate within the substantia nigra pars compacta (Marinelli, et al., 2003). It has been shown that HU210, as a synthetic cannabinoid receptor agonist, block capsaicin-induced entry of Ca^2+^. The repressive effects of this synthetic agonist, in total, are in line with the results of cannabinoid blockage of capsaicin-evoked effects (Oshita, et al., 2005).

CB1 and TRPV1 are detected in several brain areas (Fogaça et al., 2012; Mezey, 2000; Sagar et al., 2004; Tóth, Blumberg, & Boczán, 2005). There are functional relationships between CB1 and TRPV1 receptors in neuronal cells (Hermann et al., 2003; Kim, et al., 2009; Sagar et al., 2004). Both CB1 and TRPV1 receptor properties are modified by a variety of factors, including desensitization/ internalization, heterodimerization, and phosphorylation (Hudson et al., 2001; Varga et al., 2006) and are involved in neural activity. Anandamide-associated construction might act on TRPV1(di Marzo, et al., 1998; Beltramo and Piomelli, 1999).

Stimulation of CB1 receptors might heighten activity by inhibiting TRPV1 receptors (Fogaca et al., 2012). Interaction between these two systems has also been reported. One mechanism for interactive effects with CB1 regarding TRPV1 is that arousal of TRPV1 increases intracellular Ca^2+^ levels, which can trigger the Ca^2+^-related N-acyltransferase (NAT) (Reddy, et a., 1983). NAT regulates the rate-limiting stage in anandamide production and consequently, TRPV1 arousal may result in elevated anandamide production (Tóth et al., 2009). Anandamide could then trigger TRPV1 either directly or indirectly. Within the instance of direct arousal, anandamide may bind to and activate TRPV1. Direct arousal obviously happens once anandamide level is high. Low levels of anandamide may selectively trigger CB1 receptors (Tóth et al., 2009). Stimulation of CB1 receptors can result in desensitization, blockage, or potentiation of TRPV1 channels in neurons co-expressing CB1 receptors and TRPV1 channels (Hermann et al., 2003; Jeske et al., 2006; Evans, Scott, & Ross, 2007; Kim, et al., 2009; Patwardhan et al., 2006). Co-administration of CPZ and WIN possibly modifies calcium influx, release of neurotransmitters, and potentiates the anxiolytic-like response in rats.

In conclusion, our experiments showed that acute neuropharmacological stimulation of CB1 receptors or blockade of TRPV1 receptors produced anxiolytic activity. The synergic effect of CB1 receptor activation and blockade of the TRPV1 receptor potentiated their anxiolytic response. Endogenous cannabinoid signaling is possibly enhanced by co-administration of WIN and CPZ. The results of changing cannabinoid system and regulating vanilloid transmission suggest that these two networks might contribute in some common signal transduction pathways. It has been revealed that the antagonists of vanilloid system regulate cannabinoid products, which can have anxiolytic-like effects. Further studies of the interaction between the cannabinoid and vanilloid systems are warranted.
